# Antidepressant Use and the Risk of Major Adverse Cardiovascular Events in Patients Without Known Cardiovascular Disease: A Retrospective Cohort Study

**DOI:** 10.3389/fphar.2020.594474

**Published:** 2020-12-10

**Authors:** Ha Young Jang, Jae Hyun Kim, Yun-Kyoung Song, Ju-Young Shin, Hae-Young Lee, Yong Min Ahn, Jung Mi Oh, In-Wha Kim

**Affiliations:** ^1^ College of Pharmacy and Research Institute of Pharmaceutical Sciences, Seoul National University, Seoul, South Korea; ^2^ College of Pharmacy, Catholic University of Daegu, Gyeongsan-si, South Korea; ^3^ School of Pharmacy, Sungkyunkwan University, Suwon, South Korea; ^4^ Department of Internal Medicine, Seoul National University Hospital, Seoul, South Korea; ^5^ Department of Psychiatry, Seoul National University Hospital, Seoul, South Korea

**Keywords:** antidepressive agents, serotonin uptake inhibitors, tricyclic antidepressive agents, serotonin and norepinephrine reuptake inhibitor, major adverse cardiovascular events, Korean atherosclerotic cardiovascular disease risk score

## Abstract

**Aims:** Conflicting data exist on whether an association exists between antidepressants and the risk of major adverse cardiovascular events (MACEs) in patients with depression. This may be due to the use of various study designs and residual or unmeasured confounding. We aimed to assess the association between antidepressant use and the risk of MACEs while considering various covariates, including severity of depression and the cardiovascular disease (CVD) risk score.

**Methods:** Patients newly diagnosed with depression with no history of ischemic heart disease and stroke were followed-up from 2009 to 2015. We conducted Cox proportional hazard regression analysis to estimate hazard ratios (HRs) for each antidepressant for MACE risk.

**Result:** We followed-up (median, 4.4 years) 31,830 matched patients with depression (15,915 antidepressant users and 15,915 non-users). In most patients (98.7%), low-dose tricyclic antidepressants (TCAs) were related with a significantly increased risk of MACEs [adjusted HR = 1.20, 95% confidence interval (CI) = 1.03–1.40]. Duration response relationship showed a gradually increasing HR from 1.15 (95% CI = 0.98–1.33; <30 days of use) to 1.84 (95% CI = 1.35–2.51; ≥365 days of use) (*p* for trend <0.01). High Korean atherosclerotic CVD risk score (≥7.5%) or unfavorable lifestyle factors (smoking, alcohol intake, and exercise) were significantly associated with MACEs.

**Conclusion:** Even at low doses, TCA use was associated with MACEs during primary prevention. Longer duration of TCA use correlated with higher HR. Careful monitoring is needed with TCA use in patients with no known CVD history.

## Introduction

Cardiovascular disease (CVD) and depression are currently the two most common causes of disability in high-income countries (World-Health-Organization, 2008). Many studies have reported that antidepressants can increase the CVD risk. Of these, some were conducted in patients with no CVD history (primary prevention) and some in those with underlying (secondary prevention) CVD. However, no randomized clinical trials (RCTs) exist on primary prevention, due to a low CVD incidence and long follow-up durations (Oh et al., 2014). Therefore, a well-designed real world study with a sufficiently large sample size would be more appropriate. Patients with depression have a known severity of depression and CVD risk. Therefore, controlling for these confounding factors is important for a valid study design.

To our knowledge, no available observational study in the primary prevention setting has adjusted for known severity of depression and CVD risk as confounding factors while using a sufficiently large sample size. Though the application of CVD risk score is highly recommended for the primary prevention of CVD in patients without CVD history (Goff et al., 2014), many studies had no access to physical examination data [blood pressure and serum cholesterol level (Oh et al., 2014)] due to the nature of their data sources. One study included each participants’ CVD risk score (Framingham risk score) as a covariate; however, the results were based on the patients’ ability to recall, which might be subject to recall bias (Rosenberg et al., 2010). Another study could not directly calculate CVD risk scores but used the number of cardiology visits instead (Huang et al., 2013). Many studies have evaluated depression severity using the depression severity code only (Coupland et al., 2011; Scherrer et al., 2011), depression index score (Rosenberg et al., 2010; Hamer et al., 2011), and number of depression diagnoses (Blanchette et al., 2008).

We aimed to examine whether antidepressant use was associated with the risk of CVD in patients with no known CVD by considering various covariates, including depression severity and CVD risk scores.

## Methods

### Study Design and Data Sources

This study used a cohort study design and analyzed health insurance data officially provided by the Korean National Health Insurance Service (KNHIS) (Cheol Seong et al., 2017). The insurance data included the patients’ demographic, diagnosis, procedure, and prescription data. Additionally, physical examination data were linked to the KNHIS data. Physical examination data included blood sample, anthropometric measurement (blood pressure), body mass index (BMI), smoking status, alcohol consumption, and exercise data. Causes of death information was also gathered using Korean National Statistics. The requirement for written informed consent from participants was waived because all participants were anonymized by a randomized identification number. This study was approved by the institutional review board of Seoul National University (IRB No. E1606/003-002).

### Study Data

We performed analysis using KNHIS data from 2006 to 2015. Patients diagnosed with depression between 2006 and 2008 were identified using the International Statistical Classification of Diseases and Related Health Problems 10th Revision (ICD-10). Patients who had been diagnosed with ischemic heart disease and stroke between 2006 and 2008 were excluded. Physician-diagnosed depression was defined by codes F06.3, F31.3, F31.4, F32, F33, F34.1, F38.1, and F41.2. Ischemic heart disease and stroke were identified by codes I20–I25 and I60-I64, respectively. We excluded patients with the following characteristics: age <40 or ≥80 years (the suitable age range for measuring Korean atherosclerotic cardiovascular disease [ASCVD] risk scores) at the time of enrollment (Goff et al., 2014; Jung et al., 2015); cancer diagnosis (C00-C97); no history of physical examination within 1 year before the index date; prescribed antidepressants before the depression diagnosis; and did not continue initial antidepressant treatment for at least 4 weeks.

### Exposure Data

All patients’ medication information (drug name, dosage, instruction, and period) was extracted. The medications were based on the anatomical therapeutic chemical (ATC) classification system. Antidepressants were grouped into three classes (tricyclic antidepressants [TCAs]; selective serotonin reuptake inhibitors [SSRIs]; and serotonin and norepinephrine reuptake inhibitors [SNRIs]). Individuals were classified to one of the three classes based on their first antidepressant and analyzed by the intention-to-treat method. Patients with no history of antidepressant use were included in the non-user group. We used the “proportion of days covered” method to evaluate each individuals’ adherence and defined a patient as having discontinued antidepressants if the gap between prescription refills (permissible gap) was >7 days (Choudhry et al., 2009). Each daily dose was calculated by multiplying the number of tablets to be taken each day by the dose of each tablet, and this was converted to defined daily dose (DDD) which is assigned by the World Health Organization’s Collaborating Center (WHOCC) for Drug Statistics Methodology (www.whocc.no/atc_ddd_index) (WHOCC, 2016), and weighted mean DDD was calculated. Individuals’ DDD was categorized as low (<0.5 DDD), intermediate (0.5–1.0 DDD), and high (≥1.0 DDD).

### Study Endpoints (Major Adverse Cardiovascular Events)

Individuals were followed-up until 2015, and outcomes were recorded between each individuals’ index date and 2015. For CVD outcome, MACEs were used as the primary endpoints: myocardial infarction (MI) (I21), stroke (I60-I64), and CVD (I00-I99) related death. The use of invasive or surgical procedures during hospitalization for MI and stroke was additionally considered for validation (Yeom et al., 2015). Stroke was classified as hemorrhagic (I60-I62), ischemic (I63), or other (I64).

### Confounding Variables

The following baseline characteristics, potentially influencing the study outcomes, were included: age (at enrollment); sex; economic status (assessed based on income-related insurance payment); comorbidities (dyslipidemia, diabetes, and hypertension); and concomitant medications (statins, antidiabetic, and antihypertensive) within one year of the index date. Furthermore, we collected information on exercise, smoking status, and alcohol consumption from questionnaire data and blood pressure, cholesterol level, and BMI from physical examination data. Each patient’s Korean ASCVD risk scores were included as a covariate. Patients’ age, sex, total cholesterol level (mg/dl), high density lipoprotein (mg/dl), systolic blood pressure (mmHg), diastolic blood pressure (mmHg), high blood pressure treatment, diabetes diagnosis, and smoking status was used to calculate the Korean ASCVD risk score as described previously (Jung et al., 2015). For the information regarding depression severity, we used the number of outpatient visits during the first 6 months (180 days) as a proxy measure (Meunier et al., 2014).

### Statistical Analysis

To address confounding due to treatment indications, the propensity-score matching method was applied. Matching was performed using SAS 9.4 (SAS Institute Inc., Cary, NC, United States) Greedy 5→1 Digit Match macro (Parsons, 2001). The propensity score was obtained using logistic regression analysis to predict the class of antidepressants from age, sex, index year, economic status, comorbidities, co-medications, exercise, smoking status, alcohol consumption, the Korean ASCVD risk score, and the number of outpatient visits during the first 180 days after the index date. Distribution of patients’ baseline covariates was evaluated with a standardized difference. Standardized difference of <0.1 was considered indicative of good balance (NCSS-statistical-Software, 2017).

To construct the outcome model, Cox proportional hazard regression was used to estimate the hazard ratio (HR) of each antidepressant for MACE risk, with 95% confidence interval (CI). Confounding factors were exercise, alcohol consumption, BMI, number of outpatient visits, and the Korean ASCVD risk score. The proportional hazards assumption was tested graphically and confirmed for each covariate using the log(-log) plot of hazard functions for each group and covariate. Subgroup analyses were performed to investigate the risk of MACEs by age, exercise status, smoking status, alcohol consumption, BMI, and the Korean ASCVD risk category (≥7.5%, high risk category; <7.5%, low risk category) (Goff et al., 2014).

To test the robustness of our model, sensitivity analyses were performed in three ways. First, we used cancer death as a negative control in our analysis. Second, we changed the predefined gap between prescription refills and checked whether the results were influenced by medication adherence. In our original study design, if the gap between prescription refills was >7 days, the patient was considered to have discontinued antidepressant use. To prevent the patients’ medication adherence from affecting the main outcomes, we changed this gap to 14 days and 50% of each prescription period to test the effect on the main outcomes. Additionally, if the date of antidepressant initiation differed from the time of depression diagnosis, patients would have periods during which MACEs could not have been affected by treatment (immortal time) (Suissa, 2008). Therefore, we excluded patients meeting this criterion to minimize the immortal time bias. All analyses were performed with SAS software version 9.4 (SAS Institute Inc.)

## Results

**FIGURE 1:**
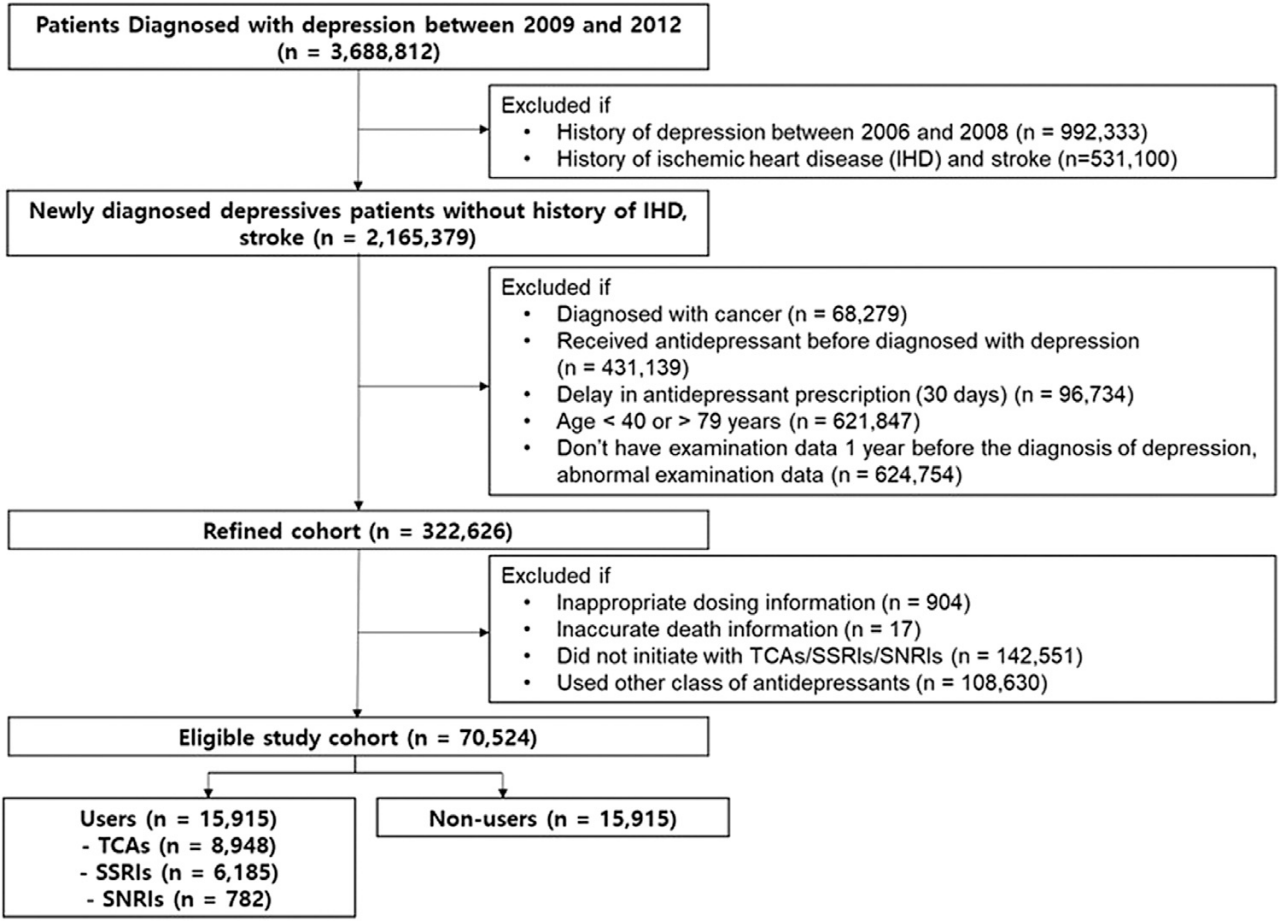
Study flow chart. TCAs, tricyclic antidepressants; SSRIs, selective serotonin reuptake inhibitors; SNRIs, serotonin-norepinephrine reuptake inhibitors.

Among 3,688,812 patients diagnosed with depression between 2009 and 2012, we excluded 1,523,433 patients owing to illness history (Figure 1). Patients not meeting the inclusion criteria or with wrong dosing information were excluded. The eligible study cohort included 70,524 patients (before propensity-score matching: 21,476 users and 49,048 non-users). Antidepressant users took more medications (statins, antidiabetics, and antihypertensive), had more comorbidities (dyslipidemia, diabetes mellitus, and hypertension), visited clinics more frequently, and had higher Korean ASCVD risk scores than non-users (Supplementary Table S1).

After propensity score matching, 15,915 antidepressant users were matched with 15,915 non-users. The above difference (comedications, comorbidities, number of clinic visit, Korean ASCVD risk scores) was reduced, and both groups were well balanced. Standardized differences were well below 0.1 for all covariates (Table 1). Median length of follow-up (4.4 years overall); median duration of antidepressants prescription during follow-up [94 (interquartile range 40–568) days]; and mean age of patients [56.4 years; men: 40.1% (*n* = 12,791)] were shown. Almost every patient (98.7%) had used low-dose TCAs (<0.5 DDD) while SSRIs were frequently used in both high (55.1%) and intermediate (43.1%) doses. Intermediate doses of SNRIs were the most frequently used (60.5%) (Table 2).

**TABLE 1:** Characteristics of major adverse cardiovascular event-free patients diagnosed with depression after propensity-score matching.

Characteristics	Non-user	User				STD
	(*n* = 15,915)	TCAs	SSRIs	SNRIs	All	
		(*n* = 8,948)	(*n* = 6,185)	(*n* = 782)	(*n* = 15,915)	
Sex (male)	6,409 (40.3)	3,622 (40.5)	2,445 (39.5)	315 (40.3)	6,382 (40.1)	−0.01
Age (year)	56.6 ± 9.8	57.2 ± 9.5	54.7 ± 10.0	56.0 ± 9.9	56.2 ± 9.8	−0.05
Economic status^a^	3.3 ± 1.5	3.2 ± 1.5	3.3 ± 1.5	3.3 ± 1.5	3.2 ± 1.5	0.05
Medication						
Statins	3,127 (19.7)	1,681 (18.8)	920 (14.9)	142 (18.2)	2,743 (17.2)	0.06
Antidiabetics	2094 (13.2)	1,154 (12.9)	436 (7.1)	90 (11.5)	1,680 (10.6)	0.08
Antihypertensive	6,828 (42.9)	3,621 (40.5)	2,428 (39.3)	293 (37.5)	6,342 (39.9)	0.06
Comorbidities						
Dyslipidemia	5,211 (32.7)	3,052 (34.1)	1,661 (26.9)	259 (33.1)	4,972 (31.2)	0.03
Diabetes mellitus	3,176 (20.0)	1893 (21.2)	871 (14.1)	148 (18.9)	2,912 (18.3)	0.04
Hypertension	5,676 (35.7)	3,030 (33.9)	1713 (27.7)	236 (30.2)	4,979 (31.3)	0.09
History of smoke/Current smoker	2,719 (17.1)	1,485 (16.6)	1,048 (16.9)	154 (19.7)	2,687 (16.9)	−0.01
Number of outpatient visits^b^	1.1 ± 0.4	1.1 ± 0.5	1.3 ± 0.6	1.2 ± 0.6	1.2 ± 0.6	0.07
BMI (kg/m^2^)	23.9 ± 3.1	24.0 ± 3.1	23.7 ± 3.1	23.6 ± 3.0	23.9 ± 3.1	0.01
Drink (time/week)	0.8 ± 1.5	0.8 ± 1.5	0.8 ± 1.5	0.9 ± 1.5	0.8 ± 1.5	0.005
Exercise (time/week)	1.0 ± 1.7	0.9 ± 1.7	1.0 ± 1.7	0.9 ± 1.6	1.0 ± 1.7	0.004
Korean ASCVD score (%)	7.4 ± 7.6	7.5 ± 7.6	6.2 ± 7.2	7.2 ± 8.0	7.0 ± 7.5	−0.05

Values are represented as mean ± standard deviation or number (%); TCAs, tricyclic antidepressants; SSRIs, selective serotonin reuptake inhibitors; SNRIs, serotonin-norepinephrine reuptake inhibitors; STD, standardized difference.

a Economic status was assessed based on income-related insurance payment.

b The number of outpatient visits during the first six months was used as a proxy measure for severity of depression.

**TABLE 2:** Doses prescribed by antidepressants class in defined daily doses.

Defined daily dose (DDD)	Class		
	TCAs	SSRIs	SNRIs
<0.5 DDD	8,832 (98.7)	109 (1.8)	174 (22.3)
0.5–1.0 DDD	105 (1.2)	2,664 (43.1)	473 (60.5)
≥1.0 DDD	11 (0.1)	3,412 (55.1)	135 (17.2)
Total	8,948	6,185	782

Values are represented as number (%); TCAs, tricyclic antidepressants; SSRIs, selective serotonin reuptake inhibitors; SNRIs, serotonin-norepinephrine reuptake inhibitors.

### Association With the Major Adverse Cardiovascular Events Component

In composite MACEs endpoint, the average time to onset of the first MACEs was 410, 409 and 301 days for the TCAs, SSRIs, and SNRIs, respectively. Only the TCAs showed a significantly increased risk of MACEs [adjusted HR (aHR) = 1.20, 95% CI = 1.03–1.40] (Table 3). The SSRIs and SNRIs were not significantly associated with the composite MACEs. When examining each MACE component, TCAs significantly increased the HR of stroke (aHR = 1.21, 95% CI = 1.01–1.44). In subtypes of stroke, only ischemic stroke was significantly associated with TCAs (aHR = 1.23, 95% CI = 1.00–1.51). SSRIs showed no significant effect on MACE risk. SNRI was a significant risk factor for MI (aHR = 3.16, 95% CI = 1.49–6.69) and CVD-related death (aHR = 2.39, 95% CI = 1.20–4.80).

**TABLE 3:** Hazard ratios for major adverse cardiovascular events (Maces) components according to the classes of antidepressants in patients without cardiovascular disease diagnosed with depression.

Class	Events	Person-year	Hazard ratio (95% CI)	
			Unadjusted	Adjusted
Major adverse cardiovascular events				
Non-users	1,173	211,744	—	—
TCAs	780	116,899	1.21 (1.03–1.41)	1.20 (1.03–1.40)
SSRIs	426	79,795	0.96 (0.80–1.17)	1.07 (0.88–1.30)
SNRIs	81	9,993	1.47 (0.99–2.17)	1.37 (0.92–2.02)
Myocardial infarction				
Non-users	159	213,581	—	—
TCAs	129	118,255	1.47 (0.99–2.20)	1.45 (0.97–2.17)
SSRIs	51	80,605	0.86 (0.50–1.48)	0.95 (0.55–1.64)
SNRIs	24	10,089	3.23 (1.53–6.79)	3.16 (1.49–6.69)
Stroke				
Non-users	921	212,013	—	—
TCAs	618	117,155	1.22 (1.02–1.45)	1.21 (1.01–1.44)
SSRIs	342	79,845	0.99 (0.80–1.22)	1.09 (0.88–1.35)
SNRIs	45	10,005	1.04 (0.62–1.74)	0.96 (0.57–1.61)
Hemorrhagic stroke				
Non-users	270	210,460	—	—
TCAs	150	116,125	1.01 (0.71–1.42)	1.00 (0.71–1.42)
SSRIs	81	79,300	0.80 (0.52–1.22)	0.86 (0.56–1.33)
SNRIs	9	9,929	0.71 (0.22–2.23)	0.67 (0.21–2.11)
Ischemic stroke				
Non-users	666	211,438	—	—
TCAs	456	116,782	1.24 (1.01–1.52)	1.23 (1.00–1.51)
SSRIs	261	79,692	1.04 (0.81–1.33)	1.16 (0.90–1.48)
SNRIs	36	9,985	1.17 (0.64–2.05)	1.06 (0.59–1.90)
CVD-related death				
Non-users	222	213,929	—	—
TCAs	141	118,568	1.15 (0.80–1.66)	1.14 (0.79–1.64)
SSRIs	105	80,701	1.26 (0.84–1.89)	1.45 (0.97–2.18)
SNRIs	27	10,103	2.60 (1.3–5.19)	2.39 (1.20–4.80)

Hazard ratio was adjusted for exercise, alcohol consumption, body mass index, the number of outpatient visits, and the Korean atherosclerotic cardiovascular disease risk score. CI, confidence interval; TCAs, tricyclic antidepressants; SSRIs, selective serotonin reuptake inhibitors; SNRIs, serotonin-norepinephrine reuptake inhibitors.

### Risk of Major Adverse Cardiovascular Events According to Antidepressants Dose and Duration

Only low TCA doses (<0.5 DDD) were significantly associated with an increased MACE risk (aHR = 1.19, 95% CI = 1.02–1.40) (Table 4). In terms of duration of use, only the duration of TCA use tended to be proportional to the MACE risk. TCA use <30 days showed no significant association with MACEs; however, with prolonged TCA use, the HR gradually increased from 1.15 (95% CI = 0.98–1.33) (<30 days of use) to 1.84 (95% CI = 1.35–2.51) (≥365 days) (*p* for trend <0.01). The HR was significantly increased in the group receiving SSRIs for 180–365 days (aHR = 1.40, 95% CI = 1.02–1.90), and there was a trend for an increasing HR in the SSRI users (0.91, 1.09, 1.40, 1.23), although the trend was not significant (*p* for trend = 0.40). There was an increased HR in patients receiving SNRIs for <30 days (aHR = 2.12, 95% CI = 1.50–3.00), and there was a trend (though not significant) for a reduction in HR (2.12, 1.27, 0.77) with an increasing duration of use (*p* for trend = 0.34).

**TABLE 4:** Hazard ratios for major adverse cardiovascular events (MACEs) according to dose and duration of each antidepressant class in patients without cardiovascular disease diagnosed with depression.

	Events	Person-years	Hazard ratio (95% CI)	
			Unadjusted	Adjusted
Dose				
Non-users	1,173	211,744	—	—
TCAs (<0.5 DDD)	774	115,304	1.21 (1.03–1.42)	1.19 (1.02–1.40)
TCAs (0.5–1.0 DDD)	6	1,428	0.64 (0.16–2.56)	2.19 (0.54–8.81)
TCAs (≥1.0 DDD)	0	167	—	—
SSRIs (<0.5 DDD)	12	1,407	1.65 (0.62–4.43)	1.33 (0.49–3.58)
SSRIs (0.5–1.0 DDD)	195	33,956	1.09 (0.84–1.42)	0.89 (0.65–1.21)
SSRIs (≥1.0 DDD)	219	44,432	1.04 (0.81–1.34)	0.86 (0.64–1.15)
SNRIs (<0.5 DDD)	18	2,303	1.18 (0.53–2.64)	1.08 (0.40–2.90)
SNRIs (0.5–1.0 DDD)	51	6,145	1.47 (0.90–2.39)	0.93 (0.48–1.81)
SNRIs (≥1.0 DDD)	12	1,545	1.28 (0.48–3.44)	0.63 (0.20–1.98)
Duration				
Non-users	1,173	211,744	—	—
TCAs (<30 days)	204	33,639	1.10 (0.94–1.27)	1.15 (0.98–1.33)
TCAs (31–180 days)	492	75,955	1.17 (1.05–1.30)	1.18 (1.06–1.31)
TCAs (180–365 days)	42	4,445	1.71 (1.25–2.32)	1.37 (1.01–1.87)
TCAs (≥365 days)	42	2,860	2.65 (1.95–3.61)	1.84 (1.35–2.51)
SSRIs (<30 days)	93	20,213	0.83 (0.67–1.03)	0.91 (0.74–1.13)
SSRIs (31–180 days)	270	51,701	0.94 (0.83–1.08)	1.09 (0.95–1.25)
SSRIs (180–365 days)	42	5,145	1.47 (1.08–2.01)	1.40 (1.02–1.90)
SSRIs (≥365 days)	21	2,736	1.39 (0.90–2.13)	1.23 (0.80–1.90)
SNRIs (<30 days)	33	3,020	1.97 (1.39–2.79)	2.12 (1.50–3.00)
SNRIs (31–180 days)	45	5,963	1.37 (1.01–1.84)	1.27 (0.94–1.71)
SNRIs (180–365 days)	0	554	—	—
SNRIs (≥365 days)	3	456	1.18 (0.38–3.68)	0.77 (0.25–2.40)

Hazard ratio was adjusted for exercise, alcohol consumption, body mass index, the number of outpatient visits, and the Korean atherosclerotic cardiovascular disease risk score. CI, confidence interval; DDD, defined daily dose; TCAs, tricyclic antidepressants; SSRIs, selective serotonin reuptake inhibitors; SNRIs, serotonin-norepinephrine reuptake inhibitors.

### Sensitivity Analyses

After the change from 7 to 14 days and 50% permissible gap, similar results were obtained (Supplementary Table S2). TCAs were still significantly associated with an increased MACE risk but SSRIs were not. An unstable result was observed for SNRIs, showing inconsistency in their level of significance for the results of each gap (7 days: aHR = 1.37, 95% CI = 0.92–2.02; 14 days: aHR = 1.49, 95% CI = 1.02–2.19; and 50% proportion: aHR = 1.25, 95% CI = 0.83–1.90). While analyzing cancer death as a negative control, all three classes showed neutral effects on MACEs (Supplementary Table S3). While testing for immortal time bias, we excluded 512 patients who had gaps between the index date and the first antidepressant exposure time. Similar results persisted for all three antidepressant classes (Supplementary Table S4).

### Subgroup Analyses

**FIGURE 2:**
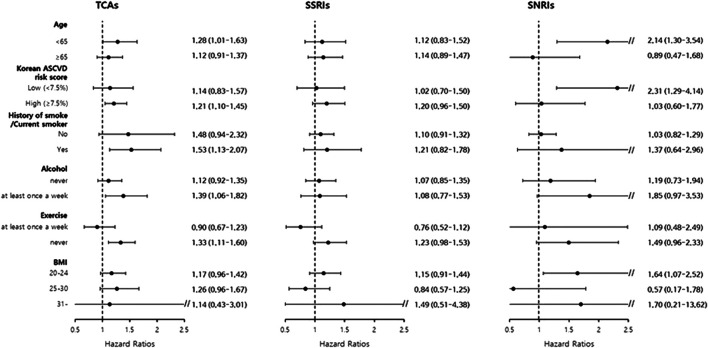
Subgroup analysis of hazard ratio for major adverse cardiovascular events based on patients’ age, exercise status, smoking status, alcohol consumption, BMI, and Korean atherosclerotic cardiovascular disease risk score category. TCAs, tricyclic antidepressants; SSRIs, selective serotonin reuptake inhibitors; SNRIs, serotonin-norepinephrine reuptake inhibitors; ASCVD, atherosclerotic cardiovascular disease; BMI, body mass index.

In TCAs, while patients aged <65 years had significantly increased risk (aHR = 1.28, 95% CI = 1.01–1.63), no effect was seen on MACE risk in those aged >65 years (aHR = 1.12, 95% CI = 0.91–1.37) (Figure 2). However, patients in the high Korean ASCVD risk group (≥7.5%) showed significantly increased risk (aHR = 1.21, 95% CI = 1.10–1.45) unlike the low Korean ASCVD risk group (aHR = 1.14, 95% CI = 0.83–1.57). We also found that patients with unfavorable lifestyle factors (smoking, alcohol consumption, and exercise) had significantly increased risk (history of smoke/current smoker = 1.53, 95% CI = 1.13–2.07; drinking alcohol at least once a week = 1.39, 95% CI = 1.01–1.82; and no exercise = 1.33, 95% CI = 1.11–1.60).

## Discussion

Our study analyzed patients with no known CVDs that were diagnosed with depression through a long follow-up study. To our knowledge, this is the first study to consider the underlying disease condition/severity and physical examination data while using a sufficiently large sample size. In addition to the previous studies reporting on the harmful effect of TCAs, new information was provided by this study on the effects of the dose/duration of antidepressants. Our study results suggest that low doses of TCA use were associated with an increased risk of MACEs. A longer duration of TCA use correlated with a higher risk.

We found significant associations between antidepressant use, particularly TCA use, and MACEs in patients with depression. However, these associations were not found with SSRI or SNRI use. Yet, even in previous studies, there does not seem to be any definite association reported between each antidepressant and a specific MACE component. We found that TCAs had a significant adverse effect on MACEs and stroke (ischemic stroke reported in the stroke subtype analysis), consistent with previous findings. The harmful effect of TCAs on MACEs has been reported, along with an association with MI (Lapane et al., 1995; Penttinen and Valonen, 1996; Pratt et al., 1996; Cohen et al., 2000; Hippisley-Cox et al., 2001; Tata et al., 2005; Rosenberg et al., 2010). Other studies have shown partially significant results on MACE components: Smoller *et al.* showed that TCA use was only associated with an increased risk of all-cause mortality but not with MI or stroke (Smoller et al., 2009). TCA use may be associated with elevated CVD risk (MI and stroke) but not with coronary heart disease alone (excluding stroke from CVD) (Hamer et al., 2011).

Most studies have reported that SSRIs have a non-significant effect on MACE components (Cohen et al., 2000; Sauer et al., 2003; Monster et al., 2004; Smoller et al., 2009; Rosenberg et al., 2010; Hamer et al., 2011; Huang et al., 2013) or a protective effect against MI (Sauer et al., 2001). However, two studies have reported a significant increase in MACE risk with SSRIs (Blanchette et al., 2008; Coupland et al., 2011): Blanchette *et al.* reported an increased risk of acute MI (Blanchette et al., 2008), while another study reported an association with increased risks of both MI and stroke (Coupland et al., 2011).

TCAs have known potential cardio-toxic effects (reduced heart rate variability and QT interval prolongation) that could lead to fatal MI, stroke, and sudden death (Roose et al., 1998). Preclinical studies have shown that TCAs have cardiovascular ion-channel- (Na(+), Ca(2+), and K(+)) blocking activities (Pacher and Kecskemeti, 2004). SSRIs could partly share this mechanism; moreover, they have been known to block serotonin transporters (Hergovich et al., 2000; Zahradnik et al., 2008). Therefore, SSRIs have often been linked to bleeding complications like gastro-intestinal bleeding or hemorrhagic stroke (Dalton et al., 2003). Thus far, the pathogenesis appears to be multifactorial such that each MACE component cannot be said to be related to a specific drug class. Discrepancies between findings may be due to the differences in study samples, study designs, or statistical methods.

In our study, the analysis of the dose and duration of antidepressants use is noteworthy. Most TCA prescriptions (98.7%) in our study were <0.5 DDD. A study reported a proportion of 70.0% which is less than that in our study (Coupland et al., 2011). According to the WHOCC, the DDDs of TCAs are 75 mg/day for amitriptyline and nortriptyline and 100 mg/day for imipramine and clomipramine (WHOCC, 2016). Most of the participants in our study used <0.5 DDD of TCAs, meaning that they used <37.5 mg/day for amitriptyline and nortriptyline or <50 mg/day for imipramine and clomipramine. In other studies, a suitable low dose was suggested in the range of 75–100 mg/day as a way to reduce side effects (Furukawa et al., 2003; NICE-Guidance, 2010). The dose used by the participants in our study was less than 75–100 mg. It seems that psychiatrists might prescribe low doses of TCAs to minimize its known harmful effects on CVDs while preserving its clinical effect on depression. Kim *et al.* reported that it is common for psychiatrists in Korea to prescribe antidepressants in doses less than the minimum effective daily dose due to their side effects (Kim et al., 2019). Our results showed that TCA use was associated with an increased risk of MACEs even at its low doses, <0.5 DDD. In the duration analysis, a longer duration of TCA use correlated with a higher HR for the MACEs. The HR when TCAs were used for more than 365 days was 1.5 times the HR when they were used less than 30 days. In addition, the average time to onset of the first MACEs was 410 days for the TCAs. Considering that the long duration of time (>365 days) and the average occurrence time of MACEs (410 days) are similar in terms of time, it would be recommended that psychiatrists monitor the occurrence of MACEs when patients use TCAs for more than 1 year. Like our study, the cardiovascular side effects of a long-term therapy (≥53 weeks) with TCAs have also been reported (Rodstein and Oei, 1979). Nevertheless, another study reported that the duration of TCA use was not correlated with MI or stroke (Coupland et al., 2016). However, in the study design, the estimated risk was calculated by dividing the exposed and unexposed periods within each patient, meaning that it was not based on their continuous use of TCAs. Unlike the method in Coupland *et al*, we estimated the risk of MACEs based on the duration of the continuous TCA use and showed the elevated risk of long term use of TCAs. Therefore, careful monitoring is needed in patients using TCAs for a long period.

In the subgroup analysis, an unusual finding was found in the age analysis. TCAs showed a significantly higher risk in younger patients (<65 years) than in older patients (≥65 years). However, in the subgroup analysis by Korean ASCVD risk scores, patients with high Korean ASCVD risk scores (≥7.5%) showed a significantly increased risk. Although the age of patients is the most powerful factor in calculating the CVD risk (Karmali et al., 2014), age alone could not be used to estimate the CVD risk of each patient. Based on these results, it is recommended that physicians consider the CVD risk score, not age alone, of each patient when prescribing antidepressants (especially when TCAs are used). We also found that patients who were former- or current smokers, who drink alcohol, and do not exercise had an increased risk of MACEs compared to the others.

There are several limitations in our study. During applying an exclusion criteria, two criteria excluded quite many people (age <40 or age ≥80: 621,847 patients; did not take physical examinations within 1 year from the index date: 624,754 patients). One of the main purposes in this study was to calculated the Korean ASCVD risk scores of patients around each of their index dates. It was inevitable that we ended up excluding a large number of people who did not have a physical examination. SNRIs had only small proportion (*n* = 782) of the matched cohort, and unstable results were obtained in their sensitivity analysis. Interpretation of the clinical meaning of the SNRIs was difficult although they were observed as a significant risk factor for MI and CVD related death. In a real world setting, there would be a large number of switches between antidepressants drugs. We excluded patients if they did not stay with their initial antidepressant classes. Therefore, we could not consider every switch between the classes of antidepressants. Our study is a retrospective cohort design and not all information is included in the KNHIS data. Therefore, although we adjusted for all possible confounders, there still might be residual confounding factors present. This study may not have completely ruled out the effects of depression on MACEs because it used number of outpatient visits as an indirect measure of depression severity.

Our study results suggest that low doses of TCA use were associated with an increased risk of MACEs in primary prevention compared with other antidepressants. A longer duration of TCA use was correlated with a higher risk. The dose and duration of antidepressants use need to be considered when TCAs are used in patients with no known CVD. High Korean atherosclerotic CVD risk score and unfavorable lifestyle factors showed significant associations with MACEs. Because no RCT evidence is available, our findings could be used when physicians prescribe antidepressants. Further research is needed to elucidate the specific mechanism and clinical significance of our study results.

## Data Availability Statement

Data that can view all the records of a patient are difficult to share due to the policy of the National Health Insurance Service. It can only be viewed in anonymized form when analyzed. Therefore, if there is a request for original data, the statistical data obtained after the desired statistical processing on the server will be shared.

## Ethics Statement

The studies involving human participants were reviewed and approved by the institutional review board of Seoul National University (IRB No. E1606/003-002). Written informed consent for participation was not required for this study in accordance with the national legislation and the institutional requirements.

## Author Contributions

HJ and JK contributed to conception and design of the study, data acquisition, analysis and interpretation of results, drafted, and revised the manuscript; Y-KS contributed to conception and design of the study, data acquisition, and revised the manuscript. J-YS contributed to design of the study, analysis of results, and revised the manuscript. H-YL and YA contributed to conception of the study, interpretation of results, and revised the manuscript. JO and I-WK contributed to conception and design of the study, analysis and interpretation of results, and revised the manuscript.

## Funding

This work was supported by the Korea Health Technology R&D Project through the Korea Health Industry Development Institute (KHIDI), funded by the Ministry of Health & Welfare, Republic of Korea (HC15C1045).

## Conflict of Interest

The authors declare that the research was conducted in the absence of any commercial or financial relationships that could be construed as a potential conflict of interest.
