# Supplementary material for: Antidepressant Use and the Risk of Major Adverse Cardiovascular Events in Patients Without Known Cardiovascular Disease: A Retrospective Cohort Study
Source: Front Pharmacol. 2020 Dec 10;11:594474. doi: 10.3389/fphar.2020.594474 (PMC7758770; doi:10.3389/fphar.2020.594474)
Supplement: Supplementary file 1 [file table1.docx]

**Supplementary Material Table 1** Characteristics of major adverse cardiovascular events-free patients diagnosed with depression before matching on propensity scores

| **Characteristics** | **Non-User**  **(n = 49048)** | **User** | | | | **STD** |
| --- | --- | --- | --- | --- | --- | --- |
|  |  | **TCAs**  **(n = 11576)** | **SSRIs**  **(n = 8823)** | **SNRIs**  **(n = 1077)** | **All**  **(n = 21476)** |  |
| Sex (Male) | 19665 (40.1) | 4658 (40.2) | 3457 (39.2) | 424 (39.4) | 8539 (39.8) | -0.01 |
| Age (year) | 55.4 ± 9.8 | 57.5 ± 9.5 | 54.9 ± 10.1 | 56.1 ± 9.8 | 56.3 ± 9.8 | 0.09 |
| Economic status^a^ | 3.2 ± 1.5 | 3.2 ± 1.5 | 3.4 ± 1.5 | 3.3 ± 1.5 | 3.3 ± 1.5 | 0.06 |
| Medication | | | | | | |
| Statins | 7037 (14.4) | 2384 (20.6) | 1413 (16.0) | 213 (19.8) | 4010 (18.7) | 0.12 |
| Antidiabetics | 3873 (7.9) | 1774 (15.3) | 728 (8.3) | 147 (13.7) | 2649 (12.3) | 0.15 |
| Antihyperten  -sives | 17032 (34.7) | 4906 (42.4) | 3675 (41.7) | 433 (40.2) | 9014 (42.0) | 0.15 |
| Comorbidities | | | | | | |
| Dyslipidemia | 12706 (25.9) | 4145 (35.8) | 2511 (28.5) | 382 (35.5) | 7038 (32.8) | 0.15 |
| Diabetes mellitus | 6946 (14.2) | 2747 (23.7) | 1345 (15.2) | 226 (21.0) | 4318 (20.1) | 0.16 |
| Hypertension | 14075 (28.7) | 4115 (35.6) | 2545 (28.9) | 348 (32.3) | 7008 (32.6) | 0.09 |
| History of  Smoke  /Current smoker | 8215 (16.8) | 1969 (17.1) | 1486 (16.8) | 208 (19.3) | 3663 (17.1) | 0.01 |
| Number of outpatient visit^b^ | 2.1 ± 2.8 | 4.4 ± 4.9 | 5.7 ± 4.8 | 3.7 ± 3.0 | 4.9 ± 4.8 | 0.72 |
| BMI (kg/m^2^) | 23.8 ± 3.0 | 23.9 ± 3.1 | 23.7 ± 3.1 | 23.7 ± 3.1 | 23.8 ± 3.1 | 0.12 |
| Drink  (time/week) | 0.9 ± 1.5 | 0.8 ± 1.5 | 0.8 ± 1.4 | 0.9 ± 1.5 | 0.8 ± 1.5 | 0.04 |
| Exercise (time/week) | 0.9 ± 1.5 | 0.9 ± 1.7 | 1.0 ± 1.7 | 0.9 ± 1.6 | 1.0 ± 1.7 | 0.03 |
| Korean ASCVD score (%) | 6.5 ± 7.2 | 7.8 ± 7.6 | 6.3 ± 7.3 | 7.2 ± 7.7 | 7.2 ± 7.6 | 0.12 |

Values are represented as mean ± standard deviation or number (%); TCAs, tricyclic antidepressants; SSRIs, selective serotonin reuptake inhibitors; SNRIs, serotonin-norepinephrine reuptake inhibitors; STD, standardized difference

^a^Economic status was assessed based on income-related insurance payment.

^b^The number of outpatient visit during the first 6 months was used as a proxy measure for severity of depression.

|  | **Events** | **Person-years** | **Hazard ratio (95% CI)** | |
| --- | --- | --- | --- | --- |
|  |  |  | **Unadjusted** | **Adjusted** |
| ***Permissive 14 days of gap*** | | | | |
| Non-users | 1323 | 227474 | - | - |
| TCAs | 882 | 128594 | 1.18 (1.02-1.37) | 1.19 (1.02-1.37) |
| SSRIs | 426 | 84298 | 0.87 (0.72-1.05) | 0.98 (0.81-1.19) |
| SNRIs | 84 | 9916 | 1.46 (1.00-2.14) | 1.49 (1.02-2.19) |
| ***Permissive 50% proportion of gap*** | | | | |
| Non-users | 1197 | 205987 | - | - |
| TCAs | 741 | 113345 | 1.12 (0.96-1.32) | 1.16 (1.00-1.36) |
| SSRIs | 420 | 77892 | 0.93 (0.77-1.12) | 1.05 (0.87-1.28) |
| SNRIs | 72 | 9815 | 1.26 (0.84-1.90) | 1.25 (0.83-1.90) |

**Supplementary Material Table 2** Hazard ratios for major adverse cardiovascular events (MACEs) according to the classes of antidepressants in MACEs-free patients diagnosed with depression after applying different permissible gaps: 14 days, 50% proportion of gap

Hazard ratio was adjusted by exercise, alcohol consumption, body mass index, the number of outpatient visits, and the Korean atherosclerotic cardiovascular disease risk score.

TCAs, tricyclic antidepressants; SSRIs, selective serotonin reuptake inhibitors; SNRIs, serotonin-norepinephrine reuptake inhibitors

**Supplementary Material Table 3** Hazard ratios for cancer death according to the classes of antidepressants in major adverse cardiovascular events-free patients diagnosed with depression

|  | **Events** | **Person-years** | **Hazard ratio (95% CI)** | |
| --- | --- | --- | --- | --- |
|  |  |  | **Unadjusted** | **Adjusted** |
| Non-users | 510 | 213929 | - | - |
| TCAs | 258 | 118568 | 0.92 (0.71-1.19) | 0.90 (0.69-1.16) |
| SSRIs | 132 | 80701 | 0.69 (0.50-0.96) | 0.76 (0.55-1.07) |
| SNRIs | 21 | 10103 | 0.88 (0.41-1.87) | 0.88 (0.41-1.88) |

Hazard ratio was adjusted by exercise, alcohol consumption, body mass index, the number of outpatient visits, and the Korean atherosclerotic cardiovascular disease risk score.

TCAs, tricyclic antidepressants; SSRIs, selective serotonin reuptake inhibitors; SNRIs, serotonin-norepinephrine reuptake inhibitors

**Supplementary Material Table 4** Hazard ratios for major adverse cardiovascular events (MACEs) according to the classes of antidepressants in MACEs-free patients diagnosed with depression after excluding 512 patients who have gap between index date and time of the first antidepressant exposure

|  | **Events** | **Person-years** | **Hazard ratio (95% CI)** | |
| --- | --- | --- | --- | --- |
|  |  |  | **Unadjusted** | **Adjusted** |
| Non-users | 1173 | 211744 | - | - |
| TCAs | 753 | 114447 | 1.19 (1.01-1.39) | 1.19 (1.01-1.39) |
| SSRIs | 411 | 76237 | 0.97 (0.80-1.18) | 1.09 (0.90-1.32) |
| SNRIs | 75 | 9516 | 1.42 (0.95-2.13) | 1.32 (0.88-1.99) |

Hazard ratio was adjusted by exercise, alcohol consumption, body mass index, the number of outpatient visits, and the Korean atherosclerotic cardiovascular disease risk score.

TCAs, tricyclic antidepressants; SSRIs, selective serotonin reuptake inhibitors; SNRIs, serotonin-norepinephrine reuptake inhibitor
